# Use of integrated genomic surveillance by local public health authorities: Recommendations based on a mixed-methods study of current adoption, applications and success factors, Germany, 2023

**DOI:** 10.2807/1560-7917.ES.2025.30.13.2400508

**Published:** 2025-04-03

**Authors:** Anna Bludau, Alexander Jack, Nicole Fischer, Johannes Dreesman, Christian Drosten, Richard Egelkamp, Lutz Ehlkes, Fabian Feil, Adam Grundhoff, Hajo Grundmann, Pascal Kreuzer, Masyar Monazahian, Inga Overesch, Daniel Schmitt, Markus Tröger, Alexandra von Reiswitz, Jonas Weber, Alexander Dilthey, Claudia Hornberg, Sandra Reuter, Simone Scheithauer

**Affiliations:** 1 Department of Infection Control and Infectious Diseases, University Medical Center Göttingen, Georg August University Göttingen, Göttingen, Germany; 2 Bielefeld University, Medical School OWL, Sustainable Environmental Health Sciences, Bielefeld, Germany; 3 Institute of Medical Microbiology, Virology and Hygiene, University Medical Center, Hamburg-Eppendorf, Hamburg, Germany; 4 Public Health Agency of Lower Saxony, Hanover, Germany; 5 Institute of Virology, Charité Universitätsmedizin Berlin, Berlin, Germany; 6 Düsseldorf Health Authority (Gesundheitsamt Düsseldorf), Düsseldorf, Germany; 7 Leibniz Institute of Virology, Hamburg, Germany; 8 Institute for Infection Prevention and Control, Medical Center - University of Freiburg, Freiburg, Germany; 9 Sozialbehörde - Amt für Gesundheit, Hamburg, Germany; 10 Institute of Medical Microbiology and Hospital Hygiene, University Hospital Düsseldorf, Heinrich-Heine-University Düsseldorf, Düsseldorf, Germany; * These authors contributed equally to this work and share first/last authorship.

**Keywords:** genomic surveillance, public health, pandemic preparedness, infection prevention and control, capacity building

## Abstract

**Background:**

Integrated genomic surveillance (IGS), i.e. the integrated analysis of pathogen whole genome sequencing and classical epidemiological data, can contribute substantially to the disease surveillance and infection prevention activities of local public health authorities (LPHAs).

**Aim:**

Our aim was to characterise how LPHAs use IGS, and factors required or important for their implementation, in the context of the German public health system.

**Methods:**

We employed a mixed-methods design combining a quantitative survey of 60 LPHAs in three German states with five qualitative case studies based on LPHAs in four German localities and one state-level public health authority.

**Results:**

Approximately half of LPHAs reported adoption of IGS; applications included outbreak analysis (n = 25), targeting and evaluation of infection control measures (n = 25 and n = 18, respectively) and characterisation of pathogen transmission chains (n = 25). Factors identified as required or important for the implementation of IGS in LPHAs included fast sample-to-result turnaround times, organisational data interpretation capabilities and clearly defined surveillance sampling strategies. Based on the case studies in which the adoption of IGS was successful, we formulate recommendations for implementing IGS at the level of LPHAs, including establishment of dedicated IGS analysis teams within LPHAs, use of user-friendly digital solutions (e.g. browser-based dashboards) for data exchange and analysis, and implementation of IGS in collaboration with local academic institutions.

**Conclusion:**

Our analysis paves the way for increasing the implementation of IGS by LPHAs in Germany and other countries with similarly structured public health systems.

**Key public health message**

**What did you want to address in this study and why?**

The COVID-19 pandemic showed the importance of genomically characterising pathogens to better understand how diseases spread and to improve prevention strategies. Local public health authorities play a key role in tracking infections, so their ability to use these methods is crucial. We wanted to assess the knowledge, needs and expectations of German public health authorities regarding genomic surveillance, and aimed to offer recommendations.

**What have we learnt from this study?**

Our research shows that many local public health authorities lacked the knowledge, resources or opportunities to use genomic surveillance effectively. Key issues such as when to use it, how to handle samples, data interpretation, digital tools, responsibilities, communication, and funding were identified. We have proposed recommendations to address these challenges.

**What are the implications of your findings for public health?**

By offering recommendations and best practices, we provide a roadmap for expanding and fully using genomic surveillance in German local public health authorities. University medical centres, especially infection prevention departments, can be ideal partners in closing this gap—not only for pandemic preparedness but also for improving infection prevention and control practices overall.

## Introduction

In many countries, local public health authorities (LPHAs) — i.e. the public health departments of municipal or district-level local governments, often staffed with around a dozen to a few hundred employees — carry the primary responsibility for monitoring and preventing the spread of infectious diseases in the community. Key tools employed by LPHAs comprise case interviews, contact tracing, surveys, as well as molecular and culture-based diagnostic testing. When available, LPHAs may also employ pathogen whole genome sequencing (WGS) approaches [1-3]. The integrated analysis of pathogen WGS and classical epidemiological data are referred to as integrated genomic surveillance (IGS).

During the COVID-19 pandemic, many countries implemented large-scale pathogen genome sequencing, contributing to the use of IGS in LPHAs and enabling, for example, the investigation of severe acute respiratory syndrome coronavirus 2 (SARS-CoV-2) transmission chains in daycare and nursing facilities [4], universities [5], industrial workplaces and commercial farms [6,7], as well as the general community [8,9]. In addition, many examples show the successful implementation of IGS by LPHAs in the context of bacterial outbreaks [1,10,11].

Based on our interactions with German LPHAs before and during the pandemic, we hypothesised that, despite its proven utility and relevance of IGS, its adoption by LPHAs may exhibit considerable heterogeneity. We are not aware of any previous attempts to systematically characterise the use of IGS in LPHAs, neither in Germany nor elsewhere. We therefore set out to study the adoption and applications of IGS at the level of the individual LPHA in the context of Germany and to identify key factors that permit, prevent or enable its implementation in LPHAs. Importantly, our study focused on how individual LPHAs apply IGS in the context of infectious disease surveillance and control in the local community. Other important applications, such as the detection of food-borne pathogen outbreaks that often involve a geographically widespread pattern of cases and which are therefore typically investigated by state-level or national public health agencies, were beyond the scope of this study.

## Methods

We employed a mixed-methods design, combining a quantitative survey targeting LPHAs in three German states with four case studies describing the use of IGS by individual LPHAs in four German localities and one case study describing the perspective of a German state-level public health authority on the adoption of IGS in LPHAs.

Our study was conducted in the context of the MolTraX project, which was the public health-focused component of the main pandemic response network of Germany’s 36 university hospitals (*The Network of University Medicine (NUM)*). The overarching aim of MolTraX, carried out between 1 July 2022 and 31 December 2023, was to characterise and support the use of genomic surveillance by German LPHAs. An important part of MolTraX was the interaction, carried out at the local level and methodologically adapted to local circumstances such as the existing degree of IGS adoption, between individual academic institutions and health authorities from the same city or region. Based on these interactions, all MolTraX participants were invited to contribute a case study on the use of genomic surveillance by the LPHAs from their corresponding regions. We included all contributed case studies in our study.

### Quantitative survey

In cooperation with the Public Health Agency of Lower Saxony (NLGA), we conducted an anonymous online survey between 22 June and 16 August 2023, aiming to quantitatively characterise the current fields of application of IGS by German LPHA as well as associated perceived challenges and opportunities. We did not calculate a sample size since we did not have any hypothesis we wanted to test. The full survey (German original and English translation) is appended in the Supplement.

For validation, the questionnaire was sent to three external experts (LPHA employees in federal states not targeted by our survey or the case studies). The experts were asked to complete the questionnaire and provide feedback on the structure, comprehensibility and relevance of the questions included in the questionnaire. Based on the experts’ responses to the questions and their feedback, the questionnaire was finalised after applying slight modifications.

Invitations were sent by email via the state offices in Lower Saxony, North Rhine-Westphalia and Hamburg to individuals responsible for the field of infection protection in all LPHAs in the respective states. We selected the three states based on their different population densities and the resulting differences in LPHA structure (e.g. different density of residents, different scope of services and specialisation, different opportunities for collaboration with other LPHAs and stakeholders).

We descriptively analysed the data using IBM SPSS 26. We conducted a descriptive subgroup analysis by state, work experience in a LPHA, and experience in dealing with WGS results. No significance was tested as the subgroups were too small for well-founded analyses. A subset of questions were only shown to the participants who had handled results from WGS in the past 5 years. In one question, participants could choose and rank their three most important framework conditions: Rank 1 resulted in 3 points, rank 2 in 2 points and rank 3 in 1 point.

### Qualitative case-studies

We complemented the quantitative survey with five in-depth case studies (drawn from the set of MolTraX participants), four of which characterised the current and potential use of IGS by the LPHAs in the corresponding two urban (Hamburg, Düsseldorf) and two more rural (Freiburg/Emmendingen, Ostwestfalen-Lippe (OWL)) localities with a particular focus on experiences during the COVID-19 pandemic. The fifth case study (Public Health Agency of Lower Saxony (NLGA)) focused on the perspective of a state-level German public health authority on the use of IGS in LPHAs.

Each case study employed its own methodological approach, adapted to local circumstances as well as to the existing degree of local IGS adoption. Briefly, the Freiburg/Emmendingen case study was based on semi-structured interviews with 14 public health professionals (seven physicians, two previous employees involved in contact tracing, two public health inspectors, one biologist and two administrative personnel) at two different LPHAs. The OWL case study was based on semi-structured interviews with 20 public health professionals from different departments and organisational levels of seven LPHAs in the OWL region in the context of a regional public health networking initiative [12]. Each of the seven LPHAs were represented by at least one physician and one person responsible for the field of infection protection. The Hamburg and Düsseldorf case studies were based on an analysis of the experiences with operating large-scale SARS-CoV-2 surveillance programmes in the two cities [13,14]. The NLGA case study was based on an analysis of the experience of bacterial outbreak investigations primarily during the COVID-19 pandemic and in the post-pandemic period. Full descriptions of the five case studies, the methods used, and the interview questions are appended in the Supplement.

To synthesise the results of the individual case studies, we employed a non-formal descriptive approach, identifying points that appeared across multiple case studies or that we deemed to be of critical importance for the successful implementation of IGS based on our subject matter expertise. Identification of these points included participation of project participants in multiple workshops, during which common and contrasting themes and categories were characterised.

## Results

### Quantitative survey

We invited 110 LPHAs to participate in the survey and received 60 complete responses (Table 1), corresponding to a response rate of 55.5% (60/110; North Rhine-Westphalia: 37/56, Lower Saxony: 23/47, Hamburg: 0/7). In six instances, the survey was started but not completed; these incomplete responses were not included in the analysis.

**Table 1:** Respondent characteristics, quantitative survey on use of integrated genomic surveillance, Germany, June–August 2023 (n = 60)

Respondent characteristic	n	%
State		
North Rhine-Westphalia (total: 56 public health agencies)	37	61.7
Lower Saxony (total: 47 public health agencies)	23	38.3
Hamburg (total: 7 public health agencies)	0	0
Age (in years)		
< 31	3	5.0
31–45	15	25.0
46–65	42	70.0
> 65	0	0.0
Profession (multiple answers possible)		
Senior physician public health services	17	28.3
Senior physician IPC, hygiene and environmental medicine	3	5.0
Senior physician with another specialisation	15	25.0
Not senior physician in IPC, but advanced course in IPC^a^	2	3.3
Junior physician	2	3.3
Public health inspector	10	16.7
Hygiene engineer	4	6.7
Medical/physician assistant	2	3.3
Degree in health science/public health/epidemiology	3	5.0
Other	10	16.7
Not specified	1	1.7
Work experience in local public health agency (in years)		
< 6	23	38.3
6–15	22	37.7
16–30	10	16.7
> 30	5	8.3
Area of the public health agency		
Small region (< 20,000 inhabitants)	0	0.0
Medium-sized region (20,000–100,000 inhabitants)	36	60.0
Large region (> 100,000 inhabitants)	23	38.3
Not specified	1	1.7

IPC: infection prevention and control.

^a^ Training in Germany after which physicians from other disciplines can work in the field of hygiene and infection control in hospitals.

The majority of participants (n = 55; 91.7%) reported having been involved in at least one outbreak investigation during the past 5 years (Table 2); one third of participants were involved in more than 25 outbreak investigations.

**Table 2:** Participation in outbreak investigations and contact with results from molecular typing results in the past 5 years, Germany, June–August 2023 (n = 60)

Experience	n	%
Participation in outbreak investigations in past 5 years		
0 times	5	8.3
1–10 times	20	33.3
11–25 times	15	25.0
26–50 times	7	11.7
> 50 times	13	21.7
Handling of results from pulsed-field gel electrophoresis in past 5 years		
0 times	3	5.0
1–4 times	15	25.0
5–10 times	42	70.0
11–25 times	0	0.0
> 25 times	1	1.7
Handling of results from multilocus sequence typing in past 5 years		
0 times	46	76.7
1–4 times	12	20.0
5–10 times	0	0
11–25 times	1	1.7
> 25 times	1	1.7
Handling of results from whole genome sequencing in past 5 years		
0 times	28	46.7
1–4 times	10	16.7
5–10 times	10	16.7
11–25 times	3	5.0
> 25 times	9	15.0
Handling of results from whole genome sequencing in past 5 years (except SARS-CoV-2)		
0 times	36	60.0
1–4 times	17	28.3
5–10 times	4	6.7
11–25 times	2	3.3
> 25 times	1	1.7

SARS-CoV-2: severe acute respiratory syndrome coronavirus 2.

Participants’ exposure over the past 5 years to different molecular typing techniques for bacteria varied from 58 for pulsed-field gel electrophoresis to 14 for multilocus sequence typing. Thirty-two participants reported having handled results from WGS; eight participants handled results from WGS only in the context of SARS-CoV-2. The majority of participants with prior WGS experience reported having worked with WGS results between one and 10 times (n = 20; 62.7% of 32 participants with WGS experience, when including SARS-CoV-2, and n = 21; 87.5% of 24 participants with WGS experience, when not including SARS-CoV-2).

Within the subset of 32 participants with WGS experience (Table 3), WGS was most frequently used for outbreaks (25/32) followed by individual pathogen fine-typing (18/32) and, at a markedly lower ratio, resistance gene detection (7/32). By contrast, with respect to the desired areas of application, the areas that were available for selection in the survey were rated to be of similar importance.

**Table 3:** Areas of, expectations for, and challenges in the application of whole genome sequencing, Germany, June–August 2023 (n = 32)

	n	%
Current areas of application (multiple answers possible)		
Outbreaks	25	78.1
Individual pathogen fine-typing	18	56.3
Detection of resistance genes	7	21.9
Surveillance	5	15.6
Detection of virulence genes	3	9.4
Not specified	2	6.3
Desired areas of application (multiple answers possible)		
Outbreaks	15	46.9
Individual pathogen fine-typing	14	43.7
Surveillance	13	40.6
Detection of resistance genes	12	37.5
Detection of virulence genes	11	34.4
Not specified	4	12.5
Expectations for the application (multiple answers possible)		
More targeted infection control measures	25	78.1
Better understanding of transmission routes over space and time (easier contact tracing)	25	78.1
Verification of an outbreak	19	59.4
Evaluation and monitoring of the effectiveness of hygiene measures	18	56.3
More detailed results	16	50.0
Falsification of an outbreak	8	25.0
Support in making a diagnosis	4	12.5
Better understanding of the characteristics and structure of the pathogen	4	12.5
Simplifying work	3	9.4
Improving therapy	1	3.1
Other	1	3.1
Challenges in the application (multiple answers possible)		
Unclear definition of indication for initiating sequencing	15	46.9
Funding/payment	12	37.5
Turnaround time for results	12	37.5
Sample logistics	9	28.1
Unclear/lacking process from initiation to communication of results	8	25.0
Lack of time	7	21.9
Insufficient quality of samples	7	21.9
Interpretation of findings	6	18.8
Communication of the results	5	15.6
Missing/unknown contact person for inquiries	5	15.6
Finding a laboratory	5	15.6
Derivation of specific measures	4	12.5
Storage of samples	4	12.5
Missing/unsuitable technology and interfaces	4	12.5
Lack of support from the organisation/supervisor	3	9.4
Other	2	6.3
Not specified	4	12.5

Participants’ expectations for WGS focused on pathogen transmission and infection prevention and control (IPC) measures; frequently selected expectations included a better understanding of transmission routes over space and time (25/32), more targeted infection control measures (25/32), and the verification of an outbreak (19/32). Of note, outbreak verification was selected by more than twice as many participants as the falsification of a suspected outbreak (8/32). Diagnosis- and treatment-related expectations were only listed by a very small number of participants. Only three of the 32 respondents expected that the use of WGS would make their work easier.

A large number of different challenges concerning the application of WGS were reported, including a lack of clarity regarding definitions of indications for initiating sequencing (15/32), funding/payment (12/32), and turnaround times for results (12/32). The handling of WGS results (deriving specific measures, interpreting the findings and communicating the results) was less frequently seen as a challenge.

Participants were asked to rank framework conditions that would improve the use of WGS. Recommendations, funding and training were rated as the most important conditions (Table 4).

**Table 4:** Ranked framework conditions to enhance the usability of integrated genomic surveillance, Germany, June–August 2023 (n = 32)

Framework condition	Points
Availability of recommendations	45
Availability of funding	34
Availability of training	28
Shorter turn-around-time for results	26
Improved communication	14
Improved sample logistics	10
Technical equipment and interfaces	10
Simplified participation of non-university large-scale laboratories	8
Improved personnel resources	7
Simplified participation of non-university hospitals	3
Simplified participation of university hospitals	0

The summed scores of all participants are shown.

The descriptive subgroup analyses by federal state, work experience in a LPHA and experience with results from WGS can be found in the Supplement; these showed, for example, a trend towards lack of clarity regarding definitions of indications for initiating sequencing being perceived as a more important limitation by low-frequency users of WGS compared with high-frequency users. No significance testing was carried out due to limited sample size.

### Qualitative case studies

We carried out in-depth case studies of the current and potential use of IGS by LPHAs in five German localities (Düsseldorf, Freiburg/Emmendingen, Hamburg, OWL and Lower Saxony) comprising urban as well as rural areas, and two LPHAs that had successfully implemented large-scale SARS-CoV-2 genomic surveillance. Full results, including a breakdown by individual case study, are appended in the Supplement. In the following, we synthesise important findings focusing on points that were identified across several case studies or that play a critical role for the successful implementation of IGS.

The need for a well-defined sampling strategy, determining which and how many pathogen samples to sequence, was identified as an important factor. We identified two possible approaches to setting sampling strategies in the case studies: (i) based on national- or state-level guidelines or (ii) at the local level, i.e. issued by the individual LPHA. The lack of an established set of sampling strategy guidelines or best practices was highlighted in three case studies (Freiburg/Emmendingen, OWL, NLGA). Two case studies (Düsseldorf, Hamburg) demonstrated successful implementation of the second approach, i.e. definition of sampling strategies at the local level. In both instances, the sampling strategies were developed in collaboration with local academic institutions and involved a combination of untargeted and targeted sequencing to allow for de novo detection of transmission chains as well as targeted investigation of suspected outbreaks. We identified additional aspects including the need for a well-defined process within the LPHA for deciding which samples to include in the targeted sampling component; the potential need for participation by experienced or specifically trained LPHA staff in this process; a potential need for being able to temporarily increase the number of sequenced samples (surge sampling); and that the time window for retrospectively selecting samples for inclusion, e.g. after the detection of a suspected outbreak, is effectively constrained by the sample storage and retention capacities of diagnostic laboratories.

We identified rapid sample-to-results turnaround times as another important prerequisite for the successful implementation of IGS. In three case studies (Freiburg/Emmendingen, OWL, NLGA), rapid turnaround times were cited as an essential requirement for deriving actionable insights from IGS, and in the two case studies of the successful implementation of large-scale IGS (Düsseldorf, Hamburg), the importance of rapid turnaround times was recognised early on and then factored into the subsequent development of the large-scale surveillance systems. We identified sample logistics, in particular with respect to samples to be acquired from commercial diagnostic laboratories for surveillance purposes, the speed of sequencing, and the timeframe until sequencing results were made available to the LPHA as important factors influencing sample-to-result turnaround times. In Hamburg and Düsseldorf, optimisation of sample logistics involved setting up a regularly executed schedule for transferring samples from diagnostic laboratories to sequencing centres according to predefined criteria (e.g. daily shipments of positive samples). The use of courier services for sample logistics was mentioned as a possible option. On the laboratory side, the use of sequencing platforms with real-time data generation capabilities (e.g. Oxford Nanopore) contributed to rapid data generation in one case study. Quick provision of results was achieved through digital solutions, such as browser-based dashboards for data exchange. In the two case studies with successful implementation of large-scale IGS, data generation and turnaround times were reduced from up to 2 weeks to around 3 days (from sample arrival until the report for the LPHA) as part of ongoing process optimisation. 

Based on the case studies, we identified the critical role of data interpretation capabilities within LPHAs. Relevant dimensions of data interpretation capabilities included the ability to interpret genomic and epidemiological data in the context of suspected outbreaks, infection contexts and transmission chains, as well as knowledge about the differences between and limitations of different sequencing-based typing methods. We identified high staff turnover, as experienced during the COVID-19 pandemic, as an important challenge for the sustainable establishment of data interpretation capabilities. Successful strategies (Hamburg, Düsseldorf) to strengthen data interpretation capabilities included setting up dedicated IGS data analysis teams within the LPHAs and embedding these teams into collaborations with local academic institutions and university hospitals, which involved participation in regular joint data interpretation meetings. Organisational data interpretation capacities were also strengthened by digital tools for data interpretation and exchange, such as browser-based applications for automated identification of potential infection clusters and integration of routinely collected epidemiological information. Furthermore, the case studies also highlighted the potential of strengthening data interpretation capabilities through structured training programmes for LPHA staff; such programmes may sustain organisational data interpretation capabilities even in times of high staff turnover. To be effective, however, training programmes would need to account for the diverse educational backgrounds of LPHA staff.

Furthermore, we identified digitisation as an important factor for the implementation of IGS. The case studies highlighted a need for effective digital processes for data exchange and communication between different LPHAs (for example in the context of outbreak investigations that concern multiple LPHAs), as well as between LPHAs and commercial diagnostic laboratories. The heterogeneity of existing digital LPHA infrastructures and German data protection regulations were identified as challenges. In two case studies (Düsseldorf, Hamburg), challenges in communication and data exchange were partially overcome by developing custom software, e.g. browser-based dashboard systems in collaboration with local academic partners.

We also identified several structural factors and framework conditions important for the implementation of IGS by LPHAs. Firstly, collaboration between the individual LPHA and local academic partners was an important factor across all case studies. The interviewed LPHA staff explicitly endorsed the importance of collaboration with academic partners in two case studies (Freiburg/Emmendingen, OWL), and in two other case studies (Düsseldorf, Hamburg), the developed surveillance platforms were operated in close collaboration with academic partners, who contributed to data generation, data interpretation and software development. Secondly, the case studies demonstrated the importance of a recognition by the relevant stakeholders (including LPHA staff, local city governments and funders) that the process of introducing IGS at the level of individual LPHAs is currently by necessity stepwise, involving a degree of pragmatism and willingness to experiment. In two case studies (Hamburg, Düsseldorf), such recognition was an important prerequisite for embarking on the project of local SARS-CoV-2 surveillance in the first place. These examples also showed that the introduction of IGS at LPHAs should be best understood as a continuous development process that can lead to marked improvements over time, e.g. with respect to the number of samples processed or turnaround times. Finally, funding is an important factor for the establishment of IGS. The five case studies showed considerable heterogeneity in the funding situation for the implementation of IGS at the level of LPHAs in Germany. In two case studies (Hamburg and Düsseldorf), the implementation of large-scale surveillance would not have been possible without state-level and academic research funding that complemented the resources made available under Germany’s national SARS-CoV-2 surveillance programme.

## Discussion

Integrated genomic surveillance can contribute substantially to the disease surveillance and infection prevention activities of LPHAs. Employing a mixed methods design combining a quantitative survey with qualitative case studies, we characterised the current use of IGS by individual LPHAs in Germany and identified which key factors prevent or enable its adoption. Our quantitative survey achieved a response rate of 55% (n = 60 respondents) in two German federal states. Furthermore, the included five case studies represented a highly diverse set of socioeconomic contexts, including rural areas and Germany’s second-largest city. 

Firstly, our results confirmed that adoption of IGS by German LPHAs is highly variable; this was evident in both the quantitative survey, with half of the respondents reporting the use of WGS during the past 5 years, as well as in the five case studies, of which three had used IGS. Importantly, the set of survey respondents may have been biased towards LPHAs or individuals with past IGS experience, so that the estimated adoption rate (but not the general finding of variable adoption across German LPHAs) may represent an overestimate. There is therefore substantial scope for strengthening the disease surveillance and infection prevention capabilities of German LPHAs by increasing the adoption of IGS.

Secondly, we identified and confirmed use cases of IGS by German LPHAs, including outbreak analysis and detection, targeting and evaluation of infection control measures, and investigation of pathogen transmission routes. The identified use cases were largely consistent between the survey, the case studies and the literature [1,10,11]. Interestingly, although the impact (e.g. with respect to the implementation of mandatory IPC measures) of showing that the cases of a suspected outbreak are not closely related (falsification of an outbreak) is often as large as the confirmation of an outbreak, the survey respondents classified the former as less important. Our findings therefore suggest scope for improving awareness of the full range of the applications of IGS, including applications that reduce workload; consistent with this, only a relatively small proportion of survey participants reported that they expected that IGS would simplify their work.

Thirdly, we identified key factors required to successfully implement IGS in individual LPHAs, including low sample-to-result turnaround times, data interpretation capabilities, well-defined sampling strategies and effective digital tools. Other studies have identified the same factors [1-3,10]. That the importance of data interpretation capabilities and digital tools was only identified in the case studies may reflect the survey’s nested design, in which items concerning challenges in the application of WGS were only shown to the subset of WGS-experienced participants.

Integrating the quantitative and qualitative results of our study and with reference to literature, we formulate a set of recommendations for increasing the implementation of IGS at LPHAs (Box).

Box **Recommendations for implementing integrated genomic surveillance in local public health authorities**

**1. Prioritise flexibility and scalability**

- Approach the implementation of IGS in LPHAs as a continuous, evolving process.
- Emphasise the ongoing progress rather than aiming for immediate perfection.

**2. Define application scenarios and objectives**

- Set clear, specific goals for introducing IGS in each LPHA.
- Tailor sampling strategies to align with these goals, ensuring relevance and effectiveness.
- Follow national guidelines and expert-set standards throughout the implementation process if available.

**3. Optimise processes and timeframes**

- Aim to achieve a turnaround time shorter than 1 week.
- Improve logistics and sequencing workflows to enhance efficiency.
- Utilise real-time sequencing technologies and digitised processes for timely results.

**4. Enhance data interpretation and analysis capabilities**

- Establish a dedicated IGS analysis team within each LPHA.
- Deploy user-friendly software for data analysis, such as tools for automated detection of genetic clusters.
- Implement structured training programmes to build robust data interpretation skills across the organisation.

**5. Build supportive structures**

- Foster a culture of innovation within the organisation.
- Maintain clear communication channels to facilitate collaboration.
- Develop partnerships with local academic institutions for knowledge sharing and support.

**6. Secure funding**

- Seek funding from national, state and municipal sources to support ongoing implementation and scaling efforts.

IGS: integrated genomic surveillance; LPHA: local public health authorities.

Most importantly, we recommend that the implementation of IGS within LPHAs should be viewed as a continuous process, not as a one-off effort. The involved stakeholders should recognise and embrace the fact that the introduction of IGS at individual LPHAs requires — at least until comprehensive national-level programmes supporting the adoption of IGS become available — a degree of pragmatism, willingness to iterate and experiment, and adoption of a ‘do not let perfect be the enemy of good’ mindset. 

Furthermore, we recommend that the process of setting a sampling strategy should begin with defining which specific goals the individual LPHA wants to achieve. The strategy will vary depending on the type of application [10]. When used as a tool for retrospective outbreak investigation, a lower background sequencing rate will be required; for the de novo detection of infection chains, more untargeted sequencing is necessary. Furthermore, sampling strategies have to take into account regulations that specify under which circumstances the application of pathogen sequencing is mandatory, as well as national or expert-set guidelines, when available [15]. 

With respect to turnaround times, we recommend an ambitious target of less than 1 week to effectively enable supporting LPHA activities in e.g. outbreak detection and management. To meet such an ambitious target, we recommend continuous process optimisation such as optimising logistics and sequencing processes, considering real-time sequencing technologies and digitised processes. In a network of public health laboratories, it might be more practical to manage workflows centrally. Single-molecule, long-read sequencing technology may have an advantage in a public health setting because of its portability, but also has the potential to reduce batch sizes and turnaround times [1].

To enable the sustainable development of data interpretation and analysis capabilities, we recommend the establishment of dedicated IGS analysis teams within LPHAs and the deployment of user-friendly software to support data analysis, e.g. for the automated detection of genetic clusters. Van Goethem et al. describe in their scoping review that expertise from bioinformatics, biology, epidemiology and microbiology needs to be combined for data interpretation [10]. To grow analytic capacity, Black et al. believe that researchers must work from both ends: increase accessibility to bioinformatics and genomic analysis for non-specialists while expanding the public health workforce skilled in bioinformatics and genomic epidemiology [3]. Structured training programmes can play an important role in the development of organisational data interpretation capabilities and ensure the continuous availability of these even in times of high staff fluctuation. With regard to supporting tools, Armstrong et al. point out that the combination and joint analysis of epidemiological and phylogenetic data is simplified by tools such as Microreact, Nextstrain or Interactive Tree of Life [1].

If possible, LPHAs should implement IGS in collaboration with local academic institutions; areas of potential collaboration include: (i) the setting of sampling strategies, benefiting from complementary academic expertise; (ii) the generation of pathogen sequencing data, potentially involving large academic sequencing cores (also implemented e.g. in COG-UK [16]); (iii) joint data interpretation meetings; (iv) software development, e.g. of web-based dashboards for the analysis of infection clusters; (v) joint training programmes. Baker et al. also emphasise the importance of trust, cooperation and shared goals not only between the research community and public health providers but also policymakers and the private sector [2].

Finally, we recommend that sufficient funding for the implementation of IGS be made available, covering sequencing costs as well as software development and training. To enable the rapid development of IGS capabilities, national-level funding can be complemented with state-level or municipal funding.

Our recommendations are directly related to the Organisational Health Literacy (OHL) of LPHA; additional relevant barriers to increasing OHL could be a lack of innovation, missing resources or an insufficient cross-sectoral cooperation or networking [17]. In order to overcome these, it is essential to promote a culture of innovation within the LPHA. Furthermore, factors such as clear communication are crucial to sustainably enhance the OHL of LPHA [17,18].

Our study was limited in a number of respects. Firstly, our study was not representative and only included LPHAs from the western parts of Germany. We targeted three federal states for the survey, representing almost a third (28 million) of the German population as well as a third of local public health authorities (110/375), and including large cities (e.g. Cologne, Hanover), one of the largest metropolitan areas in Europe (Rhine-Ruhr) as well as sparsely populated/rural areas (northern Lower Saxony, eastern North Rhine-Westphalia). The case studies were drawn from a broad national research effort (MolTrax), included Germany's second largest city (Hamburg), and also a case from another German state. While not representative, the results of our study are therefore informative about the state of the adoption of IGS and the associated challenges and best practices in Germany. In future research, it would be desirable to include LPHAs from additional German states, including the eastern parts of Germany. Secondly, while a wide range of professional backgrounds were represented in our survey, we did not attempt to explicitly represent multiple professional perspectives from within the same LPHA. For future research, it would be desirable to more explicitly capture the perspectives of a wider range of professional backgrounds from within the same LPHA and allied professions. Thirdly, a substantial proportion of our results are based on or related to SARS-CoV-2. While the experiences of the COVID-19 pandemic are certainly relevant to the general implementation of IGS, the generalisability of the SARS-CoV-2-related results to other pathogens and non-pandemic times is an important goal for future research. Fourthly, we focused our analyses on the overall use of IGS by LPHAs. While important use cases such as the investigation of healthcare-associated outbreaks were implicitly covered by the survey and case studies, the formulation of a separate set of recommendations specific to these may be warranted. Fifthly, we applied a non-formal descriptive approach to synthesise the results of the individual case studies, and those studies (as well as the local-level interactions between MolTraX participants and the LPHA the studies were based on) employed different, locally adapted methodological approaches. While carrying out our study in the context of a more formalised and unified research framework would have been desirable, the development of such a framework was hindered by the heterogeneity of the included localities regarding the implementation of IGS, as well as time pressure during the pandemic, when the MolTraX project was started. Heterogeneity in the methodological approach notwithstanding, the results reported here were generally corroborated by multiple sources (quantitative survey, multiple case study). A more unified research design to qualitatively characterise the adoption of IGS by LPHAs remains as an important goal for future research. Finally, our study was carried out in a German context, and the organisation of public health systems is country-specific in many respects; studying the generalisability of our findings to other countries therefore remains a goal for future studies.

## Conclusion

We confirmed that there is substantial scope for increasing the use of IGS by German LPHAs and developed a set of empirically based recommendations for achieving this, some of which may also be applicable to other countries. Our study offers a guide for the further strengthening of the disease surveillance and infection prevention activities of LPHAs.

## Supplementary Data

685000 Supplement
