# Supplementary material for: Use of integrated genomic surveillance by local public health authorities: Recommendations based on a mixed-methods study of current adoption, applications and success factors, Germany, 2023
Source: Euro Surveill. 2025 Apr 3;30(13):2400508. doi: 10.2807/1560-7917.ES.2025.30.13.2400508 (PMC11969963; doi:10.2807/1560-7917.ES.2025.30.13.2400508)
Supplement: Supplement [file 24-00508_DILTHEY_Supplement.pdf]

Supplementary Material

This supplementary material is hosted by Eurosurveillance as supporting information alongside the article *Use of genomic pathogen surveillance by the public health service in Germany: recommendations based on results of a mixed methods survey and case studies*, on behalf of the authors, who remain responsible for the accuracy and appropriateness of the content. The same standards for ethics, copyright, attributions and permissions as for the article apply. Supplements are not edited by Eurosurveillance and the journal is not responsible for the maintenance of any links or email addresses provided therein.

Questionnaire

Original German version (a translated version can be found below)

1. Zur Person

1.1 In welchem Bundesland arbeiten Sie?

- □ Baden-Württemberg
- □ Bayern
- □ Berlin
- □ Brandenburg
- □ Bremen
- □ Hamburg
- □ Hessen
- □ Mecklenburg-Vorpommern
- □ Niedersachsen
- □ Nordrhein-Westfalen
- □ Rheinland-Pfalz
- □ Saarland
- □ Sachsen
- □ Sachsen-Anhalt
- □ Schleswig-Holstein
- □ Thüringen

1.2 Wie alt sind Sie?

- □ ≤ 30 Jahre
- □ 31-45 Jahre
- □ 46-65 Jahre
- □ ≥ 66 Jahre

1.3 Welcher Profession gehören Sie an? *(Mehrfachauswahl möglich)*

- □ Facharzt/Fachärztin für Öffentliches Gesundheitswesen

- □ Facharzt/Fachärztin für Hygiene und Umweltmedizin
- □ Facharzt/Fachärztin für Mikrobiologe, Virologie und Infektionsepidemiologie
- □ Arzt/Ärztin anderer Fachrichtungen (Angabe)
- □ Arzt/Ärztin ohne Facharztweiterbildung
- □ Curriculare Fortbildung Krankenhaushygiene
- □ Public Health
- □ Gesundheitswissenschaften
- □ Epidemiologie
- □ Gesundheitsingenieur/Gesundheitsingenieurin
- □ Hygienekontrolleur/Hygienekontrolleurin
- □ Hygienefachkraft
- □ Medizinischer Fachgestellter/Medizinische Fachangestellte
- □ Andere *(Freitext)*

1.4 Wie viele Jahre arbeiten Sie schon in einem Gesundheitsamt?

- □ ≤ 5 Jahre
- □ 6-15 Jahre
- □ 16-30 Jahre
- □ ≥ 31 Jahre

1.5 Für welche Region ist das Gesundheitsamt, in dem sie arbeiten, zuständig?

- Kleinstadt (< 20.000 Einwohner*innen)
- Mittelstadt (20.000 – 100.000 Einwohner*innen)
- Großstadt (> 100.000 Einwohner*innen)

1.6 In wie vielen Ausbruchsuntersuchungen sind Sie im Rahmen Ihrer Tätigkeiten in den vergangenen fünf Jahren involviert gewesen?

- □ 0
- □ 1-10
- □ 11-25
- □ 26-50
- □ ≥ 51

1.7 Wie häufig haben Sie im Rahmen Ihrer Tätigkeiten in den letzten fünf Jahren mit molekularbiologischen Typisierungsergebnissen aus der Pulsed-Field-Gelelektrophorese (PFGE), Multilocus-Sequenztypisierung (MLST) oder Ganzgenomsequenzierung (z. B. cgMLST) zu tun gehabt?

	0	1-4	5-10	11-25	≥26

Pulsed-Field-Gelelektrophorese (PFGE)					
Multilocus-Sequenztypisierung (MLST)					
Ganzgenomsequenzierung (z. B. cgMLST)					
Ganzgenomsequenzierung (z. B. cgMLST) **OHNE** SARS-CoV-2					

2. Nutzung der Genomischen Erregersurveillance

Hinweis: Wir beziehen uns bei allen folgenden Fragen nur auf die *Ganzgenomsequenzierung*. *Die anderen, in der vorherigen Frage aufgelisteten Methoden werden hierbei nicht einbezogen. Die folgenden Fragen beziehen sich auf* *alle Erreger* *und nicht nur auf SARS-CoV-2.*

2.1 Zu welchen Anlässen bzw. bei welchen Anwendungsbereichen ziehen Sie die Genomische Erregersurveillance dazu bzw. würden Sie diese gerne dazu einbeziehen? *(Mehrfachauswahl möglich)*

	Wird bereits einbezogen	Sollte einbezogen werden
Allgemeine Surveillance		
Ausbrüche		
Erreger-Feintypisierung		
Detektion von Resistenzgenen		
Detektion von Virulenzgenen		
Andere (Freitext)		

2.2 Nutzen Sie diese Methode bei bestimmten Erregern häufiger als bei anderen?

- □ Ja
- □ Nein

2.3 *Wenn ja,* welche?

Freitext

2.4 Warum wird die Methode bei diesen Erregern häufiger genutzt? *(Mehrfachauswahl möglich)*

- □ Fallzahl
- □ Bereits etablierte Strukturen
- □ Expertise in Deutschland
- □ Kosten-Nutzen-Verhältnis
- □ Internationale Relevanz
- □ Wissenschaftliche Bedeutung
- □ Eigenschaften des Erregers (Morbidität, Mortalität, pandemisches Potential, Übertragungspotential, Eindämmungspotential, etc.)

- ◻ Zu beantwortende Fragestellung
- ◻ Andere *(Freitext)*

2.5 Welche Erwartungen verbinden Sie mit der Genomischen Erregersurveillance für Ihre Arbeit? *(Mehrfachauswahl möglich)*

- ◻ Arbeitserleichterung
- ◻ Detailliertere Ergebnisse
- ◻ Therapieverbesserungen
- ◻ Zielgerichtete Infektionsschutzmaßnahmen
- ◻ Besseres Verständnis der Übertragungswege über Raum und Zeit (bessere Kontaktnachverfolgung)
- ◻ Unterstützung bei der Diagnosestellung
- ◻ Besseres Verständnis der Eigenschaften und des Aufbaus eines Erregers
- ◻ Bewertung und Überwachung der Wirksamkeit von Hygienemaßnahmen
- ◻ Verifizierung eines Ausbruches
- ◻ Falsifizierung eines Ausbruches
- ◻ Andere *(Freitext)*

2.6 Sind Ihnen Herausforderungen bei der konkreten Anwendung der Genomischen Erregersurveillance begegnet? *(Mehrfachauswahl möglich)*

- ◻ Ableitung spezifischer Maßnahmen aus Befund
- ◻ Befundinterpretation
- ◻ Kommunikation der Ergebnisse
- ◻ Unklare Vorgaben für eine Veranlassung der Sequenzierung
- ◻ Unklarer/lückenhafter Prozess von Veranlassung bis Ergebniskommunikation
- ◻ Fehlende/unbekannte Ansprechpersonen
- ◻ Fehlende Zeit
- ◻ Fehlende Unterstützung der Organisation/des Vorgesetzten
- ◻ Finanzierung
- ◻ Laborsuche
- ◻ Probenlagerung
- ◻ Probenlogistik
- ◻ Untersuchungsdauer
- ◻ Ungeeignete Proben
- ◻ Hohe Datenschutzanforderungen
- ◻ Fehlende/ungeeignete Technik und Schnittstellen
- ◻ Andere *(Freitext)*

2.7 Folgen Sie bei der Anwendung der Genomischen Erregersurveillance bestimmten Empfehlungen/Vorgaben?

- ☐ Ja
- ☐ Nein

2.8 *Wenn ja,* welchen?

Freitext

2.9 Hatten Sie bereits mit einem-Ausbruch bei einem oder mehreren der folgenden Erreger zu tun? (i) SARS-CoV-2; (ii) *Salmonella* Enteritidis; (iii) *Klebsiella pneumoniae*. *Bitte umranden Sie die entsprechende Antwort.*

	GES wurde bereits bei einem Ausbruch eingesetzt.	Falls GES eingesetzt wurde, hat es die Aufklärung unterstützt?	Falls GES **nicht** eingesetzt, hätte GES Ihrer Ansicht nach die Aufklärung erleichtert bzw. verbessert?
SARS-CoV-2	Ja / Nein	Ja / Nein / bedingt	Ja / Nein / bedingt
Salmonella Enteritidis	Ja / Nein	Ja / Nein / bedingt	Ja / Nein / bedingt
Klebsiella pneumoniae	Ja / Nein	Ja / Nein / bedingt	Ja / Nein / bedingt

3. Optimierte Nutzung der Genomischen Erregersurveillance

3.1 Welche Rahmenbedingungen würden die Nutzung der Genomischen Erregersurveillance verbessern? *Wählen Sie bitte 3 Rahmenbedingungen aus und sortiere diese nach ihrer Relevanz von 1-3.*

- ◻ Verbesserte Kommunikation
- ◻ Finanzierung
- ◻ Fortbildungen zur Genomischen Erregersurveillance
- ◻ Kürzere Untersuchungsdauer
- ◻ Offizielle Empfehlungen (Anwendungsbereiche, Prozesse, Interpretation, Maßnahmenableitung)
- ◻ Verbesserte Probenlogistik
- ◻ Verbesserte Personalressourcen
- ◻ Technische Ausstattung und Schnittstellen
- ◻ Erleichterte Teilnahme von Universitätskliniken
- ◻ Erleichterte Teilnahme von nicht-universitären Krankenhäusern
- ◻ Erleichterte Teilnahme von nicht-universitären Großlaboren
- ◻ Andere *(Freitext)*

4. Abschluss

4.1 Gibt es noch Aspekte zur Genomischen Erregersurveillance, die Ihnen wichtig sind, die im Fragebogen jedoch nicht genügend Aufmerksamkeit erhalten haben?

Questionnaire – Translated English version
(the original German version can be found above)

1. About the person

1.1 In which federal state do you work?

- ◻ Baden-Württemberg
- ◻ Bavaria
- ◻ Berlin
- ◻ Brandenburg
- ◻ Bremen
- ◻ Hamburg
- ◻ Hesse
- ◻ Mecklenburg Western Pomerania
- ◻ Lower Saxony
- ◻ Northrhine-Westphalia
- ◻ Rhineland Palatinate
- ◻ Saarland
- ◻ Saxony
- ◻ Saxony-Anhalt
- ◻ Schleswig-Holstein
- ◻ Thuringia

1.2 How old are you?

- ◻ ≤ 30 years
- ◻ 31-45 years
- ◻ 46-65 years
- ◻ ≥ 66 years

1.3 What profession do you belong to? *(multiple answers possible)*

- ◻ Senior physician public health services
- ◻ Senior physician for infection prevention and control, hygiene and environmental medicine
- ◻ Senior physician for microbiology, virology and infectious diseases epidemiology
- ◻ Senior physician with another specialisation
- ◻ Junior physician
- ◻ Not senior physician in IPC, but advanced course in IPC
- ◻ Degree in Public Health
- ◻ Degree in Health Science
- ◻ Degree in Epidemiology

- ◻ Health engineer
- ◻ Public Health inspector
- ◻ Infection prevention nurse
- ◻ Medical / physician assistant
- ◻ Other *(free text)*

1.4 How many years have you been working in a public health agency?

- ◻ ≤ 5 years
- ◻ 6-15 years
- ◻ 16-30 years
- ◻ ≥ 31 years

1.5 For which area is the public health authority in which you work responsible?

- Small region (< 20.000 inhabitants)
- Medium-sized region (20.000 – 100.000 inhabitants)
- Large region (> 100.000 inhabitants)

1.6 In how many outbreak investigations did you participate during the last 5 years?

- ◻ 0
- ◻ 1-10
- ◻ 11-25
- ◻ 26-50
- ◻ ≥ 51

1.7 How often have you dealt with molecular typing results from pulsed-field gel electrophoresis (PFGE), multilocus sequence typing (MLST), or whole genome sequencing (e.g., cgMLST) in the course of your activities in the last five years?

	0	1-4	5-10	11-25	≥26
Pulsed-field gel electrophoresis (PFGE)					
Multilocus sequence typing (MLST)					
Whole genome sequencing (e.g., cgMLST)					
Whole genome sequencing (e.g.,cgMLST) **without** SARS-CoV-2)					

2 Use of Genomic Pathogen Surveillance

Note: In all of the following questions, we refer only to whole genome sequencing. The other methods listed in the previous questions are not considerate here. The following questions relate to all pathogens and not just SARS-CoV-2.

2.1 For what occasions or application areas do you use genomic pathogen surveillance, or would you like to include it? *(multiple answers possible)*

	Is already being included	Should be included
Surveillance		
Outbreaks		
Individual pathogen fine-typing		
Detection of resistance genes		
Detection of virulence genes		
Other		

2.1 Do you use these methods more often for certain pathogens than for others?

- ◻ Yes
- ◻ No

2.2 *If yes,* which ones?

Free text

2.3 Why is the method used more frequently for these pathogens? *(multiple answers possible)*

- ◻ Case numbers
- ◻ Already established structures
- ◻ Expertise in Germany
- ◻ Cost-benefit ratio
- ◻ International relevance
- ◻ Scientific significance
- ◻ Characteristics of the pathogen (morbidity, mortality, pandemic potential, transmission potential, containment potential, etc.)
- ◻ Specific research question
- ◻ Other *(free text)*

2.4 What expectations do you associate with genomic pathogen surveillance for your work? *(multiple answers possible)*

- ◻ Simplifying work
- ◻ More detailed results
- ◻ Improving therapy

- ◻ More targeted infection control measures
- ◻ Better understanding of transmission routes over space and time (easier contact tracing)
- ◻ Support in making a diagnosis
- ◻ Better understanding of the characteristics and structure of the pathogen
- ◻ Evaluation and monitoring of the effectiveness of hygiene measures
- ◻ Verification of an outbreak
- ◻ Falsification of an outbreak
- ◻ Other *(free text)*

2.5 Have you encountered challenges in the specific application of Genomic Pathogen Surveillance? *(multiple answers possible)*

- ◻ Derivation of specific measures
- ◻ Interpretation of findings
- ◻ Communication of the results
- ◻ Unclear definition of indication for initiating sequencing
- ◻ Unclear/lacking process from initiation to communication of results
- ◻ Missing/unknown contact person for inquiries
- ◻ Lack of time
- ◻ Lack of support from the organization/supervisor
- ◻ Funding/payment
- ◻ Finding a laboratory
- ◻ Storage of samples
- ◻ Sample logistics
- ◻ Turn-around-time for results
- ◻ Insufficient quality of samples
- ◻ High data protection requirements
- ◻ Missing/unsuitable technology and interfaces
- ◻ Other *(free text)*

2.6 Do you follow specific recommendations/guidelines when using genomic pathogen surveillance?

- ◻ Yes
- ◻ No

2.7 *If yes,* which ones?

Free text

2.8 Have you been involved in an outbreak of one or more of the following pathogens? (i) SARS-CoV-2; (ii) *Salmonella enteritidis*; (iii) *Klebsiella pneumoniae*. Please mark the corresponding response.

	Genomic pathogen surveillance has already been deployed during an outbreak.	If genomic pathogen surveillance was deployed, did it support the clarification?	If genomic pathogen surveillance was **not** deployed, do you believe GES would have facilitated or improved the clarification?
SARS-CoV-2	Yes / No	Yes / No / conditional	Yes / No / conditional
Salmonella Enteritidis	Yes / No	Yes / No / conditional	Yes / No / conditional
Klebsiella pneumoniae	Yes / No	Yes / No / conditional	Yes / No / conditional

3 Optimized utilization of Genomic Pathogen Surveillance

3.1 What surrounding conditions would enhance the utilization of genomic pathogen surveillance? *Please choose three surrounding conditions and rank them in order of relevance from 1 to 3.*

- ☐ Improved communication
- ☐ Funding
- ☐ Training
- ☐ Shorter turn-around-time for results
- ☐ Recommendations (application areas, processes, interpretation, derivation of measures)
- ☐ Improved sample logistics
- ☐ Improved personnel resources
- ☐ Technical equipment and interfaces
- ☐ Simplified participation of university hospitals
- ☐ Simplified participation of non-university hospitals
- ☐ Simplified participation of non-university large-scale laboratories
- ☐ Other *(free text)*

4 Completion

4.1 Are there any aspects of genomic pathogen surveillance that are important to you but have not received sufficient attention in the questionnaire?

Free text

Interview guide
Original German version (a translated version can be found below)

1. Zur Person
    1.1. Wie lange arbeiten Sie schon in Ihrem Bereich?
    1.2. Wie häufig pro Jahr arbeiten Sie mit Stammtypisierungen? Sind Sie in Ausbruchsuntersuchungen involviert?
    1.3. Hatten Sie schon einmal mit Typisierungsergebnissen aus der Ganzgenomsequenzierung, Pulsed-Field-Gel-Elektrophorese (PFGE) oder Multilocus-Sequenztypisierung (MLST) zu tun?
2. Nutzung der Genomischen Erregersurveillance
    2.1. Was verstehen Sie unter Genomischer Erregersurveillance? -> Hinweis: Wir beziehen uns bei allen folgenden Fragen nur auf die Ganzgenomsequenzierung. PFGE und MLST werden hierbei nicht einbezogen
        2.1.1. Was haben Sie unter Genomischer Erregersurveillance verstanden, bevor Sie von GenSurv und MolTraX gehört haben? Oder haben Sie überhaupt darüber nachgedacht?
    2.2. Zu welchen Anlässen bzw. bei welchen Anwendungsbereichen ziehen Sie die Genomische Erregersurveillance dazu? Arbeiten Sie dabei priorisiert mit bestimmten Erregern? Warum werden diese priorisiert?
    2.3. Welche Vorteile sehen Sie dabei in der Genomischen Erregersurveillance für Ihre Arbeit?
    2.4. Können Sie den allgemeinen Ablauf vom Anlass bis zur Nutzung und Kommunikation der Ergebnisse beschreiben – gerne auch an einem konkreten, vergangenen Beispiel? Gehen Sie dabei auch gerne auf „Erfolgserlebnisse“ und Herausforderungen ein.
    2.5. Folgen Sie dabei bestimmten Empfehlungen/Vorgaben?
    2.6. Falls zuvor nicht bereits behandelt: Hatten Sie bereits mit einem größeren Ausbruch von (i) SARS-CoV-2 (ii) nicht SARS-CoV-2 zu tun? Falls ja, was waren die größten Herausforderungen bei der Aufklärung und glauben Sie, dass der Einsatz von Genomischer Erregersurveillance (falls nicht bereits erfolgt) die Aufklärung erleichtert hätte?
    2.7. Welche Rahmenbedingungen würden die Nutzung der Genomischen Erregersurveillance verbessern? (Finanzierung, Schnelligkeit, Kommunikation, Technische Voraussetzungen, …)
    2.8. Wie könnte ein optimaler Prozess für die Nutzung der Genomischen Errergersurveillance aussehen? (von Anlass bis zur Nutzung und Kommunikation der Ergebnisse)
3. Abschluss
    3.1. Gibt es noch Aspekte, die Ihnen wichtig sind, die im Interview noch nicht genügend Aufmerksamkeit erhalten haben?

Interview guide – Translated English version
(the original German version can be found above)

1. Personal details
    1.1. How long do you work in your current field?
    1.2. How often do you work with typing results per year? Are you involved in outbreak investigations?
    1.3. Have you worked with results from whole genome sequencing, pulsed-field gel-electrophoresis (PFGE) or multilocus sequence typing (MLST)?
2. Use of genomic pathogen surveillance
    2.1. What is your understanding of genomic pathogen surveillance? -> note: the following questions only relate to whole genome sequencing; PFGE and MLST are not considered.
        2.1.1. What did you know about genomic pathogen surveillance before being introduced to GenSurv and MolTraX? Have you thought about it before at all?
    2.2. In which cases do you employ genomic pathogen surveillance? Do you prioritize particular pathogens? Why are these prioritized?
    2.3. Which advantages do you see in genomic pathogen surveillance for your work?
    2.4. Can you detail the workflow from ignition to use and communication of results – do give concrete past examples. Make a mention of “success stories” as well as challenges.
    2.5. Do you follow particular recommendations or requirements?
    2.6. If not answered before: Have you been involved in a large outbreak of (i) SARS-CoV-2 (ii) not SARS-CoV-2? If yes, what were the biggest challenges in resolving the outbreak and do you think the use of genomic pathogen surveillance (if not already employed) would have helped?
    2.7. Which framework conditions would make the use of genomic pathogen surveillance better? (financing, speed, communication, technical requirements, ….)
    2.8. What would an optimal workflow for using genomic pathogen surveillance look like? (from starting event to use and communication of results)
3. End
    3.1. Are there any further aspects important to you that have not had enough attention thus far?

Description of the case studies

Düsseldorf

Background: Düsseldorf Health Authority (DHA) is responsible for covering all public health-related affairs in the city of Düsseldorf, an international economic and air travel hub of approximately 600,000 inhabitants centrally located in Germany's largest metropolitan area. During 2021, DHA successfully employed integrated genomic surveillance (IGS) for monitoring the spread of SARS-CoV-2 variants of concern (VOCs), for the retrospective investigation of putative SARS-CoV-2 outbreaks in various contexts, and for the de novo detection of population SARS-CoV-2 transmission chains and infection clusters. IGS in Düsseldorf was set up as a separate project ("Integrated Genomic Surveillance System Düsseldorf") outside of existing local public health structures and as a collaboration between DHA, Heinrich Heine University Düsseldorf (HHU), and two large commercial diagnostic labs from Düsseldorf.

Approach: Key results and learnings from the period during which the Integrated Genomic Surveillance System Düsseldorf was operated were identified and summarized in collaboration between DHA and HHU, involving key members of the DHA and HHU departments tasked with the implementation of the Integrated Genomic Surveillance System Düsseldorf.

Results: IGS enabled the tracing of hundreds of infection chains and infection clusters in the population that would otherwise have remained undetected. It furthermore supplied evidence to uncovering violations against mandatory infection prevention rules, e.g. in nightlife settings; contributed to the implementation of improved infection prevention rules e.g. in hospitals; and informed the shift from containment to protection after the introduction of the Omicron VOC (cf. National Plan for Pandemic Preparedness https://www.gmkonline.de/documents/pandemieplan_teil-i_1510042222_1585228735.pdf). Results from IGS were regularly communicated between DHA and the city's mayoral municipal leadership and informed communication towards the general public via real-time communication of VOC data on a browser-based dashboard system.

Key elements of the successful implementation of IGS in Düsseldorf included (i) rapid sequencing of a large proportion of SARS-CoV-2 cases enabled by optimized logistics and close collaboration with commercial diagnostic labs (>30% of cases between February and October 2021, with results of viral genome sequencing often becoming available a few days after the initial PCR test); (ii) application of automated algorithms for the detection of potential infection clusters in the generated genetic data;

(iii) utilization of user-friendly browser-based dashboards for data exchange between the participating institutions, including tools for the visualization and investigation of genetically identified candidate infection clusters; (iv) having a dedicated team of up to 5 specifically trained case investigators within DHA assigned to all aspects of IGS-related analyses and efforts, including retrospective outbreak analyses and investigation of genetically identified clusters; (v) support for the IGS project by DHA leadership. In a retrospective review of the IGS effort in Düsseldorf, the significant manual effort required for the investigation of genetically identified putative clusters was identified as a key challenge; in addition, improved integration with existing IT infrastructures was identified as desirable. Software that enables the automated integration of viral sequencing data with routine contact tracing data collected within the SurvNet software package used at DHA, streamlining the investigation of genetically identified infection clusters, was developed in 2022 but could not yet be tested at scale. Of note, the IGS effort in Düsseldorf benefited from pre-pandemic training and experience of DHA leadership in genomic surveillance, including participation in the Postgraduate Training for Applied Epidemiology programme offered by Robert Koch Institute.

Hamburg

Background: With over 1.9 million inhabitants, Hamburg is the second largest city in Germany. In Hamburg, during the winter of 2020/2021 the Leibniz Institute of Virology (LIV), the University Medical Center Hamburg Eppendorf (UKE) and state health authorities jointly installed a molecular SARS-CoV-2 surveillance platform (HH-SuRV; https://www.leibniz-liv.de/de/aktuelles/covid-19/daten-der-hamburg-surveillance-plattform/), which was launched in January 2021 and ran continuously for 2.5 years until April 2023. The platform performed systematic PCR- and NGS-based surveillance of SARS-CoV-2 variants, as well as continuous assessment of infection dynamics in the Hamburg metropolitan area based on molecular epidemiology techniques. Furthermore, the platform routinely assisted local public health authorities (LPHA) in the resolution of transmission chains and infection clusters (e.g., in childcare centers and workplaces[1] and contributed to the establishment of improved infection

[1] Nakel J, Robitaille A, Günther T, et al. Comparing susceptibility and contagiousness in concurrent outbreaks with a non-VOC and the VOC SARS-CoV-2 variant B.1.1.7 in daycare centers in Hamburg, Germany. Int J Hyg Environ Health. 2022;240:113928. doi:10.1016/j.ijheh.2022.113928
Pfefferle S, Günther T, Kobbe R, et al. SARS Coronavirus-2 variant tracing within the first Coronavirus Disease 19 clusters in northern Germany. Clin Microbiol Infect. 2021;27(1):130.e5-130.e8. doi:10.1016/j.cmi.2020.09.034
Brehm TT, Thompson M, Ullrich F, et al. Low SARS-CoV-2 infection rates and high vaccine-induced immunity among German healthcare workers at the end of the third wave of the COVID-19 pandemic. Int J Hyg Environ Health. 2021;238:113851. doi:10.1016/j.ijheh.2021.113851
Günther T, Czech-Sioli M, Indenbirken D, et al. SARS-CoV-2 outbreak investigation in a German meat processing plant. EMBO Mol Med. 2020;12(12):e13296. doi:10.15252/emmm.202013296

prevention measures in hospitals[2]. Overall, within the HH-SuRV framework more than 18,000 viral genomes from all phases of the pandemic were sequenced, analyzed and archived.

Approach: Key results and learnings from the period during which HH-SuRV was operated were identified and summarized in collaboration between LIV, UKE and Hamburg public health authorities, involving key members of the corresponding departments tasked with the implementation of HH-SuRV.

Results: Establishment of the HH-SuRV program was greatly facilitated by the fact that, during the years prior to the pandemic, LIV and UKE had already established a NGS-based metagenomics platform dedicated to the analysis of pathogens in primary diagnostic material. With the appearance of the first case in Hamburg at the end of February 2020, the partners thus were able to immediately start cataloging viral entries in Hamburg, with the explicit goal of tracking the spread of SARS-CoV-2 infections and the potential emergence of variants in the metropolitan region. To achieve a coverage as representative as possible, the samples were randomly selected from a pool collated to represent individual districts according to local incidence. Longitudinal analysis of mutational profiles served to detect locally expanding infection clusters and identified variants potentially emerging from the metropolitan area.

The rapid response and synergy between the scientific institutes, the university hospital and public health services were seen as very positive, particularly considering future challenges in local infection control. The partners agreed that, to meet such challenges, the established communication channels and data exchange interfaces should be maintained and further optimized. The best way forward would be a continued joint-surveillance program of recurring or potentially emerging infectious agents (e.g. influenza viruses, polio- or other enteroviruses, monkeypox or bacterial pathogens like EHEC and Salmonella) at a scale that is both epidemiologically meaningful and economically sustainable. Such a program could serve as an early warning system for health authorities, while at the same time fostering further development of public health-oriented infrastructures, e.g. via maintaining up-to-date software solutions allowing molecular epidemiology analyses. Of course, continuous data integration and exchange between local and national surveillance efforts would be of the highest importance.

[2] Czech-Sioli M, Günther T, Robitaille A, et al. Integration of Sequencing and Epidemiologic Data for Surveillance of Severe Acute Respiratory Syndrome Coronavirus 2 (SARS-CoV-2) Infections in a Tertiary-Care Hospital. Clin Infect Dis. 2023;76(3):e263-e273. doi:10.1093/cid/ciac484

The Public Health Agency of Lower Saxony (NLGA)

Background: The Public Health Agency of Lower Saxony (NLGA) is responsible for public health-related surveillance in Lower Saxony, Germany's second largest state by size or fourth largest state by population. In addition to its epidemiological expertise, it also offers lab-based diagnostics as support for LPHA. In 2018, the NLGA opened a lab for next generation sequencing with focus on bacterial cluster detection, identification of antibiotic resistance or virulence genes and species determination and *in silico* serotyping.

Approach: Pre-pandemic, the laboratory was mainly contacted when there was a strong suspicion of an outbreak in order to support the assumption by sequencing. However, this limited perception of the technical possibilities by LPHA changed with the widespread use of NGS for SARS-CoV-2 sequencing.

Results: An increasing number of LPHA are taking advantage of the services offered in order to be able to monitor even smaller potential outbreaks with NGS data, especially, as costs for LPHA outbreak investigations at the NLGA are covered by the state of Lower Saxony. In addition, some regions started with an NGS-based routine monitoring of specific pathogens, e. g. *Legionella pneumoniae* or Vancomycin-resistant *Enterococcus faecium*, to be able to identify previously undetected outbreaks or monitor long-term events. Furthermore, a multispecies outbreak with a carbapenemase-containing plasmid is currently being investigated, which so far includes seven different bacterial species. The insights gained through sequencing have already helped in the analysis of this unusual outbreak, so that further samples are now to be included in a carbapenemase-focused study.

While the increasing awareness of sequencing has brought many benefits, it has also highlighted some limitations, particularly on (i) the technical side, (ii) communication and (iii) data interpretation at the local level. A major technical hurdle here is e. g. the exchange of metadata and sequence data between different laboratories without media discontinuity, as a central German data hub for this kind of information is missing. Therefore, metadata is often sent in parallel with samples on paper, while sequence data is exchanged bilaterally via various cloud storage systems. In addition, there are several cases in which times of two or three weeks between sample collection and the NGS result, whether due to missing guidelines for an NGS-relevant sample collection, slow sample transfer between the primary laboratory and the NLGA or missing metadata, have made it difficult for the involved LPHA to react quickly. While these problems are primarily of a technical nature and are already being addressed by the institutions involved, (ii) and (iii) represent problems at a different organizational level. Many LPHA lack personnel with biomolecular and bioinformatic expertise, leading to misconceptions about the possibilities and limitations of sequencing. This further complicates local data interpretation, which is why either sometimes a sequencing request may not be made,

information is not used to its fullest or additional time-consuming communication with experts at the NLGA is necessary.

To ensure broad and efficient future use, technical exchange platforms for metadata, sequence data and interactive data interpretation must be created and financed on a permanent basis. In addition, further guidelines and training must be created specifically for employees of health agencies in order to ensure the necessary expertise for fluent communication as well as comprehensive and independent data interpretation. While first steps were already taken during the pandemic, the initial enthusiasm must not be lost in the return to everyday life, but rather maintained through targeted support and used to establish a modern genomic surveillance system.

Freiburg/Emmendingen

Background: In Baden-Wurttemberg, two LPHA were included: Landkreis Breisgau-Hochschwarzwald, which includes the city of Freiburg, and thus covers both rural and urban areas (270,000 and 235,000 inhabitants, respectively), and the neighbouring Landkreis Emmendingen (170,000 inhabitants). Most personnel joined during the pandemic, only three physicians and one hygiene inspector had been with the health authorities prior to the pandemic.
Approach: Interviews (see Supplement 2) were conducted with volunteers from both LPHAs based on two interview guidance documents, the first focusing exclusively on IGS and the second containing broader themes around pandemic handling and preparedness. Fourteen people were interviewed: seven physicians, two previous employees involved in contact tracing, two public health inspectors, one biologist, and two administrative personnel. Only four (three physicians, one public health inspector) had been employed prior to the pandemic.
Results: All interviewees had been involved in outbreak investigations, and all had used typing results, however mainly to determine variants of SARS-CoV-2, and only when necessary. Similarly, molecular typing was used to inform on variants and types, but differences in typing methods were largely unknown. Genomic surveillance became known during and through the pandemic, but was not identified as a concept as such before the survey. Genomic surveillance is mainly thought of in terms of variant typing, and to monitor contaminated food and zoonoses. Therefore, the main species for which it could be applied are food-related (Salmonella, EHEC, Listeria, Campylobacter, norovirus), as well as Streptococci, Mycobacterium tuberculosis, and multi-drug resistant bacteria. Especially with respect to food-borne investigations, genomic surveillance may be of use in detecting potential sources for outbreaks that are geographically spread and include temporarily disconnected cases. Hence, cross-sectional surveillance - monitoring animal, environment, and humans, akin to a One

Health approach - is expected to benefit most from genomic surveillance, for better connection of outbreaks. An advantage is also seen in relying less on humans to provide information; they might not be able to judge their situation, symptoms, and implications properly, and might also not be truthful in their answers towards health authorities. This might be especially beneficial in case of refugees, and has been demonstrated to an extent by tracing skin diphtheria cases along the refugee routes, and which also highlights the potential for European-wide surveillance across borders. Lastly, it is expected that genomic surveillance would provide actionable, convincing data; in cases where legal authority is needed, this might be easier to obtain with genomic data.
The main challenge identified is the need for timeliness of the genomic data and the results. There is no use for sequencing data if it is received retrospectively, at which point it is "nice to have" but does not result in any action. A sampling and sequencing strategy or recommendations are needed, as well as funding. Additionally, health authorities need quicker and more digital ways for communication between different health authorities as well as laboratories. For improved communication, central databases for contextualization of outbreaks are also seens as useful. Since knowledge on typing methods and therefore also knowledge how to judge them in terms of reliability and resolution is lacking, training of health authority personnel is seen as a key framework condition. Training is then also recommended to include interpretation and the use of genomic data. Within health authorities, people also come from different backgrounds - medical, non-medical, even non-scientific - and thus basic training in typing methods would also provide a good working basis for everyone. Another challenge particularly in Germany remains around data protection, which needs to be adapted so that effective and quick communication of relevant information between health authorities is possible, again with the help of digital, protected, and standardized software. With the generational change within the health authorities - many joined new during the pandemic - there is a good opportunity for personnel to embrace newer methods and have a higher affinity for digital solutions. Effective implementation of genomic surveillance would be helped by connections to universities and university hospitals.

Region of East-Westphalia Lippe (OWL)

Background: The region of East-Westphalia Lippe (OWL) represents a socio-demographic heterogenic area consisting of seven districts (Bielefeld, Lippe, Gütersloh, Minden-Lübbecke, Höxter, Paderborn and Herford) with over 2 million inhabitants in both rural and urban settings, respectively. The seven corresponding LPHAs are organized within a so-called MRE – network, which endorses collaboration across communal boundaries to enhance prevention against infectious diseases. The university of Bielefeld upholds a long-lasting collaboration together with this network. This intersection between

the local public health sector and academic medicine provided a suitable framework for obtaining qualitative information on various aspects regarding IGS employment by LPHAs.

Approach: Representatives of all seven LPHAs in OWL participated in the interviews which were carried out across different hierarchies. For each location, we interviewed the head of that department along with 4 health inspectors with minimum work experience of four years (in total seven physicians and 28 health inspectors). It is also worth mentioning that the interviewed persons gathered additional information from other colleagues involved in pandemic infection tracing prior to our scheduled interviews.

Results: After completing the interviews and based on the accumulated information from public health staff, we compiled the qualitative results into two categories. First, the advantageous and beneficial aspects for successful IGS from a user's point of view were grouped under promoting factors. Opposed to that, all topics that were considered as disadvantageous and problematic for sustaining IGS by LPHA staff were grouped under inhibiting factors.

Promoting factors

For successful implementation of IGS within an ongoing outbreak scenario, LPHA staff emphasized that availability of sequence data within 2-3 days has to be achieved in order to make the best use of genomic information for current outbreak management.

The most relevant factors uniformly considered by the interviewees as highly advantageous include for instance quick sample collection (surge sampling). Such tasks should ideally be performed by experienced personnel, followed by fast sample logistics preferably carried out by lab courier services. To further support the ongoing IGS process, well-established communication routes between laboratories and LPHAs were also considered as highly significant (e.g. rapid sample analysis with focus on pre-labeling isolates of interest for direct post-PCR sequencing to save crucial time while avoiding time-consuming bureaucracy lines). Additionally, public health staff should be trained to analyze and interpret genomic data along with epidemiological infection tracing information on a regular basis. As an element to support IGS for retrospective analyses of scenarios where an outbreak was not initially suspected or identified, the LPHAs reported that collaboration with labs offering sample storage capacities are considered highly beneficial.

The more of the above-mentioned factors align, the higher the chance to successfully control outbreak scenarios and minimize spreading events and hygiene gaps.

Inhibiting factors

Basically, any circumstances that oppose or counteract the aforementioned enabling aspects are considered as hurdles to establish and uphold IGS successfully. Issues that could hinder proper execution of IGS are predominantly insufficient infrastructural parameters, in particular digitalization at the LPHA as well as linkage to commercial labs with respective capacities of fast-sequencing techniques. Personnel fluctuation rates have been described as challenging by the heads of the health departments, as they lead to higher need of time-consuming coaching of new staff on a regular basis. On an administrative level, inconsistent communication of political orders (e.g. informing the public prior to health departments about new regulations) as well as often unclear SOPs or responsibilities were strikingly considered as heavy burdens posing additional challenges within the already demanding framework of outbreak control and infection tracing during the pandemic.

Subgroup analysis of the quantitative survey

	Lower Saxony		North Rhine-Westphalia	
	Total (n=10)	Percent	Total (n=21)	Percent
Current areas of application (multiple answers possible)[a]				
Outbreaks	5	55.6	20	90.9
Individual pathogen fine-typing	6	66.7	12	54.5
Detection of resistance genes	4	44.4	3	13.6
Surveillance	2	22.2	3	13.6
Detection of virulence genes	2	22.2	1	4.5
Not specified	0	0.0	1	4.5
Wanted areas of application (multiple answers possible)[a]				
Outbreaks	6	66.7	9	42.9
Individual pathogen fine-typing	7	77.8	7	33.3
Surveillance	3	55.6	7	33.3
Detection of resistance genes	4	44.4	9	42.9
Detection of virulence genes	3	33.3	8	38.1
Not specified	0	0.0	4	19.0
Expectations for the application (multiple answers possible)				
More targeted infection control measures	9	90.0	16	72.7
Better understanding of transmission routes over space and time (easier contact tracing)	8	80.0	17	77.3
Verification of an outbreak	6	60.0	13	59.1
Evaluation and monitoring of the effectiveness of hygiene measures	8	80.0	10	45.5
More detailed results	6	60.0	10	45.5
Falsification of an outbreak	2	20.0	6	27.3
Support in making a diagnosis	1	10.0	3	13.6
Better understanding of the characteristics and structure of the pathogen	2	20.0	2	9.1

Simplifying work	1	10.0	2	9.1
Improving therapy	0	0.0	1	4.5
Other	0	0.0	1	4.5
Challenges in the application (multiple answers possible)				
Unclear definition of indication for initiating sequencing	6	60.0	9	40.9
Funding/payment	4	40.0	1	4.5
Turn-around-time for results	5	50.0	7	31.8
Sample logistics	3	30.0	6	27.3
Unclear/lacking process from initiation to communication of results	3	30.0	5	22.7
Lack of time	3	30.0	4	18.2
Insufficient quality of samples	5	50.0	2	9.1
Interpretation of findings	3	30.0	3	13.6
Communication of the results	1	10.0	4	18.2
Missing/unknown contact person for inquiries	3	30.0	2	9.1
Finding a laboratory	4	40.0	1	4.5
Derivation of specific measures	4	40.0	0	0.0
Storage of samples	3	30.0	1	4.5
Missing/unsuitable technology and interfaces	1	10.0	3	13.6
Lack of support from the organisation/supervisor	1	10.1	2	9.1
Other	0	0.0	2	9.1
Not specified	0	0.0	4	18.2

Table S1: Areas of, expectations for, and challenges in the application of whole genome sequencing divided by state, Germany, survey was conducted from June to August 2023, n=32. [a] one answer is missing from Lower Saxony.

	Low frequency		High frequency	
	Total (n=10)	Percent	Total (n=9)	Percent
Current areas of application (multiple answers possible)				
Outbreaks	7	70.0	6	75.0
Individual pathogen fine-typing	8	80.0	3	37.5
Detection of resistance genes	2	20.0	3	37.5
Surveillance	0	0.0	3	37.5
Detection of virulence genes	0	0.0	1	12.5
Not specified	0	0.0	1	12.5
Wanted areas of application (multiple answers possible)				
Outbreaks	3	30.0	6	66.7
Individual pathogen fine-typing	4	40.0	7	77.8
Surveillance	5	50.0	4	44.4
Detection of resistance genes	4	40.0	7	77.8
Detection of virulence genes	4	40.0	7	77.8
Not specified	1	10.0	0	0.0
Expectations for the application (multiple answers possible)				
More targeted infection control measures	9	90.0	8	88.9
Better understanding of transmission routes over space and time (easier contact tracing)	9	90.0	8	88.9
Verification of an outbreak	5	50.0	8	88.9
Evaluation and monitoring of the effectiveness of hygiene measures	6	60.0	6	66.7
More detailed results	5	50.0	5	55.6
Falsification of an outbreak	2	20.0	4	44.4
Support in making a diagnosis	2	20.0	1	11.1
Better understanding of the characteristics and structure of the pathogen	1	10.0	1	11.1
Simplifying work	0	0.0	2	22.2
Improving therapy	0	0.0	0	0.0

Other	0	0.0	0	0.0
Challenges in the application (multiple answers possible)				
Unclear definition of indication for initiating sequencing	7	70.0	3	33.3
Funding/payment	3	30.0	6	66.7
Turn-around-time for results	3	30.0	3	33.3
Sample logistics	3	30.0	3	33.3
Unclear/lacking process from initiation to communication of results	4	40.0	3	33.3
Lack of time	1	10.0	3	33.3
Insufficient quality of samples	3	30.0	3	33.3
Interpretation of findings	1	10.0	1	11.1
Communication of the results	2	20.0	1	11.1
Missing/unknown contact person for inquiries	1	10.0	1	11.1
Finding a laboratory	1	10.0	0	0.0
Derivation of specific measures	1	10.0	1	11.1
Storage of samples	1	10.0	2	22.2
Missing/unsuitable technology and interfaces	0	0.0	1	11.1
Lack of support from the organisation/supervisor	0	0.0	0	0.0
Other	2	20.0	0	0.0
Not specified	1	10.0	0	0.0

Table S2: Areas of, expectations for, and challenges in the application of whole genome sequencing divided by frequency of handling results from molecular biological typing results, Germany, survey was conducted from June to August 2023, n=32. Low frequency = 1-4 times in the last five years, high frequency = over 26 times in the last 5 years

	Work experience ≤ 5 years		Work experience ≥ 6 years	
	Total (n=12)	Percent	Total (n=20)	Percent
Current areas of application (multiple answers possible)				
Outbreaks	10	83.3	15	75.0
Individual pathogen fine-typing	7	58.3	11	55.0
Detection of resistance genes	2	16.7	5	25.0
Surveillance	3	25.0	2	10.0
Detection of virulence genes	2	16.7	1	5.0
Not specified	1	8.3	1	5.0
Wanted areas of application (multiple answers possible)[a]				
Outbreaks	6	60.0	9	45.0
Individual pathogen fine-typing	4	40.0	10	50.0
Surveillance	4	40.0	9	45.0
Detection of resistance genes	4	40.0	8	40.0
Detection of virulence genes	5	50.0	6	30.0
Not specified	1	10.0	3	15.0
Expectations for the application (multiple answers possible)				
More targeted infection control measures	9	75.0	16	80.0
Better understanding of transmission routes over space and time (easier contact tracing)	9	75.0	16	80.0
Verification of an outbreak	7	58.3	12	60.0
Evaluation and monitoring of the effectiveness of hygiene measures	8	66.7	10	50.0
More detailed results	7	58.3	9	45.0
Falsification of an outbreak	4	33.3	4	20.0
Support in making a diagnosis	2	16.7	2	10.0
Better understanding of the characteristics and structure of the pathogen	3	25.0	1	5.0
Simplifying work	1	8.3	2	10.0

Improving therapy	0	0.0	1	5.0
Other	0	0.0	1	5.0
Challenges in the application (multiple answers possible)				
Unclear definition of indication for initiating sequencing	5	41.7	10	50.0
Funding/payment	6	50.0	6	30.0
Turn-around-time for results	7	58.3	5	25.0
Sample logistics	4	33.3	5	25.0
Unclear/lacking process from initiation to communication of results	4	33.3	4	20.0
Lack of time	4	33.3	3	15.0
Insufficient quality of samples	3	25.0	4	20.0
Interpretation of findings	3	25.0	3	15.0
Communication of the results	3	25.0	2	10.0
Missing/unknown contact person for inquiries	3	25.0	2	10.0
Finding a laboratory	1	8.3	4	20.0
Derivation of specific measures	1	8.3	3	15.0
Storage of samples	2	16.7	2	10.0
Missing/unsuitable technology and interfaces	2	16.7	2	10.0
Lack of support from the organisation/supervisor	1	8.3	2	10.0
Other	0	0.0	2	10.0
Not specified	1	8.3	3	15.0

Table S3: Areas of, expectations for, and challenges in the application of whole genome sequencing divided by frequency of handling results from molecular biological typing results, Germany, survey was conducted from June to August 2023, n=32. [a] two answers are missing from Lower Saxony.
